# Special Court Negatives the Proposed Changes

**Published:** 1913-12-27

**Authors:** 


### THE MANAGEMENT OF ST. GEORGE'S HOSPITAL.

### Special Court Negatives the Proposed Changes.

A special court of the governors of St. George's Hospital was held in the Board-room of the hospital on Friday, December 19. Sir William H. Bennett, K.C.V.O., presided over the first part of the proceedings.

Mr. J. Montague, Lord Moulton of Bank, and Sir George Hayter Chubb, Bart., were unanimously elected new governors.

### Proposal to Appoint Mr. A. W. West Secretary-Superintendent.

The court then passed to consider proposals made by the House Committee for the management of the hospital, which involved additional expenditure.

The Chairman said that the report of the finance committee of November 24 was divided into three sections, and emendations had been made in each case by the House Committee. The first dealt with the pension to be paid Mr. Wingrove upon his retirement, and the second with the salary of a resident medical superintendent. These two were dependent on, and must stand or fall with, the third, which he suggested should be taken first.

Some discussion took place on the technicalities of procedure, and the Chairman expressed the hope that the very difficult business of the court would not be hampered by technical objections. He agreed that his ruling on that occasion should not be taken as a precedent.

Mr. Warrington Haward then moved :

> That the secretary and superintendent be given a salary of £600 a year, with £150 a year for board and servants, and that he be lodged in a portion of No 9 Knightsbridge.

This being formally seconded by Captain H. Claude Hay,

Mr. Haward briefly gave his reasons for asking support for this resolution. He thought that if the resolutions arrived at by the House Committee were not confirmed, very strong reasons ought to be given for such a course, especially as the House Committee's recommendations were based upon those of a small sub-committee, specially appointed to advise them upon the management of the hospital. The Committee were unanimous in the opinion that it was desirable to retain Mr. West's services, should he be willing to accept the position of a paid official. It was recommended, therefore, that Mr. West should be invited to accept the office of secretary, with supervision of the hospital, save for that portion connected with the resident medical staff. They had arrived at a critical period of the hospital's history, and it was important to have the entire service of someone familiar with its work. For twelve years Mr. West had been intimately associated with the management of the hospital, and it would be disastrous if they lost Mr. West's services at that moment. Their secretary, excellent fellow as he was, was not competent to carry out the complicated work which was required at this juncture. If Mr. West were given the position suggested, it would not be a matter of finding a post for the treasurer, but of appointing a secretary. He knew that some present had not agreed with all that Mr. West had done. This was inevitable. Mr. West had been actuated honestly by the endeavour to do what was best for the hospital. He had saved money to the hospital, and his organising capacity had been well tested. This was not a new proposal at all. They were merely proposing to resume the arrangement which existed in Mr. Todd's time, and those who remembered it would recall its smooth working. They were told that it would mean an increase of expenditure, but that was a misapprehension. When Mr. Todd was secretary he was paid £600, the resident medical officer £450, and the assistant secretary £240—a total of £1,290. The House Committee's proposal was that the secretary should be paid £600, the resident medical officer £300, and the assistant secretary £140—a total of £1,040. There was, however, the question of the pension of the retiring secretary. It was proposed that his full salary of £300 should be paid for two years, until he reached the age of sixty, and thereafter £160 per annum. For the first two years, therefore, the total charge would be £50 more than it was in Mr. Todd's secretaryship, and afterwards it would be considerably less. He pleaded for the putting aside of all personal prejudice, and said that after fifty years' constant work in connection with the hospital he did understand a little about its needs.

### Text of the Amendment.

Mr. A. Keyser moved as an amendment that,

> The court, having duly considered the resolutions passed by the House Committee for the creation of a special highly paid post for the treasurer, involving considerable extra expense on the finances of the hospital, and entailing the resignation of their secretary at his full salary, declines to confirm them.

He said that these proposals appeared to him to provide a comfortable sum for a gentleman to do nothing in order to give another gentleman a nice salary. Their immediate effect would be to add £750 a year to the hospital expenditure. Mr. Todd was still enjoying, he was pleased to say, a large pension, and it was proposed to put in a new man on the same salary as Mr. Todd. The speaker's objections were not only financial. He quite agreed with Mr. Haward in admiring Mr. West's industry, and the very great interest he had taken in the affairs of the hospital. Further than that, he did not go. In common with many other governors, he had been far from satisfied with parts of Mr. West's administration. The first thing that caused difference among them was undoubtedly the dispute with regard to the King's Fund. On that occasion there were literally two parties, Mr. West and the medical school on the one side, and the majority of the lay governors on the other. The speaker also referred to causes of irritation in connection with the elections last June, and entered into many details. When it was made known that Mr. West could not afford to go on as treasurer without payment, the first suggestion was that there should be a paid treasurer. There proved, however, to be no other instance of a paid treasurer in London. He sketched a history of the recent negotiations, and said with regard to the suggestion to pension Mr. Wingrove, if Mr. Wingrove were competent, he ought not to be asked to go, and to give him his full pay on retirement in order to make room for an officer they wished to appoint, appeared to him (the speaker) a proposal that could not be entertained by those responsible for the funds of the hospital. The constitution of the hospital was so contrived, owing chiefly to the hasty manner in which the House Committee was substituted for the old weekly board, that the medical side had as many members as the lay, and now with four places not filled up, the laymen were in a minority. When, therefore, Mr. Haward said that these proposals had the approval of the House Committee, he thought the words "medical school" might almost be substituted. It was not the custom in other hospitals to give the gentlemen of the medical schools so large a preponderance. The speaker added that Mr. West's transactions with regard to the sale of the site had given rise to dissatisfaction, and the critical period before the hospital, and the proposed amalgamation, were to his mind an argument against retaining Mr. West's services. He asked his fellow governors to acquit him, as his conscience acquitted him, of any feeling in the matter save a desire to serve the best interests of the hospital.

Mr. R. H. C. Harrison seconded the amendment. He fully agreed with everything that Mr. Keyser had said. Until recently he had been in constant touch with Mr. West, but he must say that he found on many occasions that Mr. West had not carried out the whole of the duties that he should have done as chairman of an independent committee. He criticised the terms for the proposed sale of the site. On a governor rising to a point of order, Mr. Harrison said that he was only referring to these negotiations because of their bearing upon Mr. West's management of the hospital. He went on to refer to the smallness of the deposit—less than 2 per cent.—to be paid by Mr. Mallaby-Deeley, the prospective purchaser, and to the fact that the transaction had not taken place through the lawyers of the hospital. With regard to Mr. Haward's statement that the expenditure was not greater now than it was under Mr. Todd, he would point out that although Mr. Todd was getting £600 a year he had been through the hospital, and was at the top of the tree before he retired. Was it suggested that Mr. West should begin at the top of the tree? To his mind it would be most disastrous if it went abroad that in a hospital like theirs they had got to a state in which it was necessary to create a special post for one man in order that he should not have to resign his position among them, the alternative of not resigning being that he must give up his own private work.

### Criticism and Interruptions.

Dr. Ridge Jones, whose speech was much interrupted, said that of late years he had not been at all satisfied with the way the management of the hospital had been carried on. Important changes had taken place. An open board had been done away with and a close board had been established instead without any diminution in the number of the medical staff having votes on it. A large contribution of the hospital fund had been paid each year to the medical school, by which the treasurer had secured the support of the staff, but which caused the King's Hospital Fund to suspend contributions to the hospital. The loss of this during the last three or four years amounted to about £6,000, with the result that £4,000 more capital last year had to be sold out, making a total permanent loss of £10,000 to the hospital. These changes and altered management had resulted in practically ruining the medical school, and if the diminution of the subscriptions to the hospital continued at the same rate the donations and legacies in time would follow suit, with the result that sooner or later the hospital would also be ruined.

A Governor: If the speaker says that Mr. West's action has ruined the medical school he surely ought to give some proofs.

Dr. Ridge Jones, continuing, said that the treasurer, having been refused a salary for his services, applied to be appointed superintendent and secretary with a salary which, including house and servants, would be equivalent to about £1,200 a year, or the pay of Prince Louis of Battenberg, the First Sea Lord.

A Governor: It would not be fair to let that pass—that Mr. West applied to be secretary.

Dr. Ridge Jones thought it came to the same thing. He hoped all who wished to see that old institution survive and prosper would support the amendment.

### A Temporary Proposal.

Mr. G. R. Turner said that a great many mis-statements had been made with regard to the medical staff and the medical school, and dealt in detail with some of them. The proposal to make Mr. West a paid secretary was not meant as a permanent proposal, but only until such time as the amalgamation of the two hospitals, or the removal from their present site, was completed. At the first meeting of the sub-committee appointed to consider the future management of the hospital, it was decided that it was not advisable to consider the question of the management with any reference to the constitution, but to limit the discussion to the management of St. George's Hospital in the immediate future. Some governors felt that Mr. West in his management of the hospital had offended them. The speaker was no apologist for Mr. West, but at the same time he regarded him personally as one who knew most about the hospital, and some of them thought that when they were crossing a stream it was unwise to change horses. He protested against the statement that the medical staff wanted to have undue preponderance in the rule of the hospital.

Sir Henry Burdett asked whether Mr. West was still treasurer of the hospital, and the Chairman replied that he was. Proceeding, Sir Henry said that if the resolution were carried it would bring the hospital into such a state of internal discord as to make the administration extremely difficult. He referred to the fact that it was proposed to have a resident medical superintendent as well as a secretary-superintendent, and on the ground purely of sound management he urged that the governors of the hospital should never consent to any such proposals. During the last twenty years there had been a serious diminution in the ordinary income of St. George's Hospital. But some other Metropolitan Hospitals of similar size to St. George's, including the London Hospital, during this period had increased their ordinary income by from 25 to 125 per cent.?—[A voice: "The King's Fund."]—Sir Henry pointed out that these figures were based upon the finances of the last twenty years, long before that dispute arose. The fact was that they had not got a single man at that hospital who understood the way to raise money and get at the proper people. He should therefore vote for the amendment. He had worked with Mr. West most cordially, and recognised his services over several years. But Mr. West had not the qualifications nor had he the knowledge which the hospital required in order either to raise its revenue as it must be raised, whether or not amalgamation took place, or to administer successfully its affairs.

The Chairman then put the amendment moved by Mr. Keyser, and there voted:—

| | |
|---|---|
| For the amendment . . . . | 30 |
| Against . . . . . . . | 18 |

On putting the amendment as a substantive motion, it was carried by the same figures—30 votes to 18.

Sir W. H. Bennett then left the chair, and when his place was taken by Mr. A. W. West a large number of the governors had left the meeting.

The other resolutions relating to the pension of the secretary and the salary of the resident medical superintendent, being contingent upon the one relating to the salary of the secretary and superintendent, were formally negatived.

### The Questions of Amalgamation and Site.

A report was then read from the House Committee relative to the question of amalgamation and the purchase of a new site and incidental matters. The ground of the report was fairly covered in four resolutions subsequently submitted. Before the first of these was put, however,

The Chairman (Mr. West) said that since the report was drawn up some changes had taken place, especially with regard to the negotiations for the purchase of their present site. Owing to difficulties with the Charity Commissioners and other causes, Mr. Mallaby-Deeley had recognised the position and withdrawn his offer. Hitherto in view of these negotiations many people had hesitated to make offers, but he was assured that there were now plenty of people ready to come forward with offers.

On the proposition of Mr. Keyser, seconded by Mr. Harrison, the report of the House Committee, dated December 10, was adopted.

The Chairman said that the Westminster Hospital had a Bill of their own, which had passed, but there would be considerable difficulties about the Amalgamation Bill. Neither Westminster nor themselves had actually sold their sites, and it was thought best that they should proceed with an independent Bill.

Sir Henry Burdett asked whether they had power in the Bill to amalgamate with Westminster Hospital, and was answered in the affirmative. He was glad, then, that the scheme for amalgamation was still alive, and had not been abandoned by anybody.

Mr. Harrison moved, and Mr. Keyser seconded, and it was carried *nem. con.* :—

> 2. That the court is of opinion that it is desirable to abandon for the present Session of Parliament the joint Bill prepared with respect to the amalgamation of the hospital with Westminster Hospital, and to proceed with a Bill on behalf of St. George's Hospital alone, on the lines of the draft Bill which has been laid on the table, and approves the action which the House Committee has taken in that behalf.

#### The Wandsworth Site.

Dealing with resolution 3, the Chairman explained that if the Wandsworth Bridge site were bought it would not render it impossible ultimately to buy another site for the amalgamated hospitals, should the two hospitals agree to amalgamate. The whole staff of the Westminster Hospital was in favour of the Wandsworth Bridge site, providing no better one could be found. But both hospitals were agreed that they did not want their hands tied. There was one difficulty owing to the clause in the title which prohibited the erection of anything on the site that would be a cause of annoyance or a risk of fire to the next-door neighbours. The solicitor thought there would be no difficulty in getting a licence from the neighbours to cover this objection. The valuer said that £27,000 might be justifiably paid for the Wandsworth Bridge site if a smaller sum could not be agreed upon, and that the risk they were running in making the purchase was a matter of some £3,000, as they could re-sell the site for £24,000.

Mr. G. R. Turner said that the staffs of the two hospitals had come to the conclusion that the great thing to aim at was amalgamation. The question of site was secondary. The relative healthiness of the Wandsworth and the Clapham sites seemed to be much on a level.

Sir Henry Burdett asked the reason for an immediate decision.

Mr. Turner explained that the option to buy expired on the following morning.

Sir Henry Burdett thought it was not business to take the risk of buying a site and then to go into negotiations with tied hands. If there was a restriction against the building of a hospital on the site which had been suggested it was perfectly clear that they could not get over it. Judicial ruling was very clear upon that point.

Colonel Glyn said that if it turned out that they were not allowed to build the hospital on the site, and lost £3,000, it would be a great misfortune. He would not do business along those lines himself, nor as trustee.

After some further discussion Mr. Harrison moved, and Mr. Turner seconded, on the understanding that they were not bound of necessity to the Wandsworth site, and were not handicapped with respect to amalgamation, the third resolution, which ran :

> That the court is of opinion that it is desirable to secure a site which will be suitable either for a new joint hospital or a new St. George's Hospital, and is further of opinion that the Wandsworth Bridge site is a suitable site.

The motion was carried, with five dissentients.

When the following resolution was moved :—

> That the House Committee be directed to negotiate for the purchase of the Wandsworth Bridge site by St. George's Hospital, and be authorised to enter into such contract or contracts to be settled by Conveyancing Counsel as the House Committee shall think fit for *purchasing or* obtaining an option or options to purchase such site at a price not exceeding £27,000 (exclusive of any sums paid for options).

Sir Henry Burdett moved the omission of the words "purchasing or." As they were trustees he thought they could not go further than to sanction authority to obtain an option.

Captain Glyn, in seconding the amendment, said that he could not be a party to the purchase of a site on which it may be impossible to erect a hospital, where it may possibly be found to interfere with the proposed amalgamation, or to render possible a heavy loss on resale.

Mr. Turner said that he took it that the buying of the Wandsworth property did not commit them to it as a site. The Chairman of Westminster Hospital had acquired a portion of the site at Clapham, and it would not handicap them in future negotiations with Westminster to purchase the Wandsworth site.

Sir Henry Burdett said that if, as Mr. Turner had suggested, Westminster was hobbled with a part of a site, this was no reason, but quite the contrary, why St. George's should be hobbled with a whole site.

Sir Henry Burdett's amendment was put to the meeting, and carried, and the resolution as amended was then put as a substantive motion, and agreed to without dissent.

### An Independent Committee to Select a Site.

The Chairman reported the following resolution had been passed by the joint meeting of members of the medical staffs of the St. George's and Westminster Hospitals on December 17, 1913 :—

They would therefore recommend that if the House Committees of the two hospitals fail to agree on the question of a site, an independent committee be appointed to advise on the choice of site, one member to be nominated by the House Committee of St. George's Hospital, one member to be nominated by the House Committee of Westminster Hospital, and one member by the Speaker of the House of Commons, as representing the King Edward's Hospital Fund, and further that each House Committee should pledge itself to abide by the decision of the independent committee.

